# Sleep and Aging Across the Globe

**DOI:** 10.1093/geroni/igaf122.767

**Published:** 2025-12-31

**Authors:** Tuo Yu Chen, Soomi Lee, Orfeu Buxton

## Abstract

The increasing prevalence of poor sleep worldwide is a growing concern, given its detrimental effects on physical health, mental health, longevity, and overall quality of life. The relationship between sleep, aging, and health varies across different countries and cultures, highlighting the need to explore sleep in diverse contexts and establish standardized assessment methods. This symposium brings together four presentations that delve into sleep and aging among various populations, focusing on its implications for life and health expectancy, sociobehavioral and lifestyle risk factors, and methodological advancements in sleep research. The first study investigates the associations between sleep and nap duration with total life expectancy and cardiovascular disease-free life expectancy in older adults in Singapore, shedding light on sleep’s role in promoting healthy aging. The second study examines how work and family spillover—the interplay between two central social roles—relates to sleep quality over time among midlife adults in the US and Japan, highlighting cultural similarities and differences in occupational stress and sleep disruption. The third study explores lifestyle factors related to multidimensional sleep health among Japanese adults aged 40 years and older, as well as age-related variations in these associations. The last study focuses on innovative methodologies for assessing multidimensional sleep health, leveraging data from older cohorts in the US and the Netherlands. Finally, Dr. Orfeu Buxton will lead a discussion on the key findings from these various countries and methodological approaches, fostering a deeper understanding of sleep health’s role in aging and identifying future directions for global sleep research.

